# Computed tomography and magnetic resonance imaging approaches to Graves’ ophthalmopathy: a narrative review

**DOI:** 10.3389/fendo.2023.1277961

**Published:** 2024-01-08

**Authors:** Rafael Luccas, Cinthia Minatel Riguetto, Monica Alves, Denise Engelbrecht Zantut-Wittmann, Fabiano Reis

**Affiliations:** ^1^ Graduate Program of Neuroscience, Faculty of Medical Sciences, State University of Campinas, Campinas, Brazil; ^2^ Department of Anesthesiology, Oncology and Radiology, Faculty of Medical Sciences, State University of Campinas, Campinas, Brazil; ^3^ Division of Endocrinology, Faculty of Medical Sciences, State University of Campinas, Campinas, Brazil; ^4^ Waikato Regional Diabetes Service, Te Whatu Ora Health New Zealand, Hamilton, New Zealand; ^5^ Department of Ophthalmology and Otorhinolaryngology, Faculty of Medical Sciences, State University of Campinas, Campinas, Brazil

**Keywords:** Graves’ ophthalmopathy, Graves’ disease, imaging analysis, magnetic resonance imaging, computed tomography

## Abstract

Graves’ ophthalmopathy (GO) affects up to 50% of patients with Graves’ disease (GD) ranging from mild ocular irritation to vision loss. The initial diagnosis is based on clinical findings and laboratory tests. Orbital imaging, such as magnetic resonance imaging (MRI) and computed tomography (CT), is an important tool to assess orbital changes, being also useful for understanding disease progression and surgical planning. In this narrative review, we included 92 studies published from 1979 to 2020 that used either MRI and/or CT to diagnose and investigate GO, proposing new methods and techniques. Most of the methods used still need to be corroborated and validated, and, despite the different methods and approaches for thyroid eye disease (TED) evaluation, there is still a lack of standardization of measurements and outcome reports; therefore, additional studies should be performed to include these methods in clinical practice, facilitating the diagnosis and approach for the treatment of TED.

## Introduction

Graves’ disease (GD), the most common causes of hyperthyroidism in iodine-replete areas, is characterized by the excess of thyroid hormones associated with loss of the negative feedback between the hypothalamic–pituitary axis and the thyroid gland due to circulating thyroid-stimulating anti-thyrotropin receptor antibodies (TRAb) (1). TRAb mimic the action of hormone, excessively activating thyroid gland follicular cells and inducing its growth and vascularization (2). GD has an approximate incidence of 40 cases, each with 100,000 persons, being more frequent in women and patients between 30 and 50 years old, and may include extrathyroidal features, including Graves’ ophthalmopathy (GO) (3, 4).

GO affects up to 50% of the patients, varying from mild ocular irritation to vision loss (2). Patients may experience conjunctival redness, conjunctival and eyelid swelling, proptosis, retrobulbar pain, ocular dryness, photophobia, and reduced visual acuity secondary to optic nerve compression and cornea erosion. From a mechanical point of view, these signs and symptoms are due to an enlargement of orbital fat tissue and extraocular muscles, compressing the surrounding structures (5). Although the diagnosis of GO is based on clinical findings such as inflammation of the orbit and proptosis, imaging exams, including magnetic resonance imaging (MRI), computed tomography (CT), and ultrasonography, are useful in the identification and follow-up of clinically active GO (6) as well as unusual cases of unilateral ophthalmopathy, optic neuropathies, and optic nerve compression (7).

Imaging exams are not an essential tool to diagnose GO in clinical practice; however, they can add valuable information on tissue microstructure, leading to new findings on actual disease progress and status, which is vital for planning treatment and interventions (8). Imaging in patients with GO may reveal an increase in orbital fibroadipose tissue, enlargement of extraocular muscles, and optic-nerve compression. Imaging is especially warranted in cases of asymmetric proptosis and for differential diagnosis (9, 10).

Orbital imaging is an important tool to assess disease progression and for surgical planning. MRI and CT can be used individually or mutually for these applications. The enlargement of extraocular muscles is the main characteristic of GO. Still, it is challenging to determine if the muscle is volumetrically enlarged in the scenario of symmetric mild disease, where the patient-specific normal measure parameter is lost (4). On the other hand, there has been developed normative data that define an average for normal extraocular muscle measurements (11–14) as a guide, but not as a norm, because it represents variations and overlap cases on both healthy and diseased muscles.

The inferior rectus muscle is the most frequently involved in clinical myopathy, followed by medial, superior, and lateral rectus muscles, respectively. In GO, multiple muscles can be involved simultaneously and/or bilaterally in 76% to 90% of the cases. Extraocular muscle enlargement is the most common sign among these patients, usually affecting younger and older patients differently. According to Su et al. (15), younger patients are less affected by motility restriction despite developing more proptosis even with enlarged muscles, whereas older patients suffer from more motility restriction and diplopia due to the muscle bellies enlargement posterior in the orbit. Proptosis is mainly related to the most enlarged muscle position than the size itself; however, imaging analysis for muscle enlargement is not precise or sensitive and should be used as adjuvant evidence for diagnosis (16).

Another remarkable characteristic of GO is the fat expansion associated with proptosis, younger patients, and a longer GO duration. Visually, fat expansion can expand the brow, galeal, and premalar regions but may also be identified and measured by imaging. Both fat expansion and muscle enlargement are usually measured in volume individually.

Exophthalmos is usually measured during clinical evaluations by a Hertel exophthalmometer, but imaging techniques can be also used for this measurement, tracing a reference line between the anterior extents of each zygomatic bone in a mid-orbital axial section and then measuring the distance from this reference line to the posterior aspect of the ocular globe. A distance of 10 mm or less indicates exophthalmos (9). The level of proptosis is indicated by grades: grade 1 when more than two-thirds of the anteroposterior diameter of the eyeball is projected to the front of the line, grade 2 when the posterior pole of the globe border the line, and grade 3 when the entire eyeball is projected in front of this line (17).

Due to the difficulties in accessing GO image changes, our aim was to review systematically studies that used imaging approaches to diagnose or investigate the disease, proposing new methods and techniques or further investigating the usual ones.

## Method

The search for these studies occurred on the main databases such as PubMed, Embase, Scopus, and Web of Science in September 2021. This review used the Preferred Reporting Items for Systematic Reviews and Meta-Analyses (PRISMA) statement (18) as a reference for the article structure.

Publications with no year restriction until the search date were considered, involving CT or MRI approaches in patients with GD who did not undergo clinical or surgical treatment to evaluate clinical activity or severity of the disease. Studies without a control group were also considered because the objective of this review is also to give the full scenario of the current state of the studies and research works being done with imaging in GO and how the study designs differ from each other. Due to the large number of results generated by the search strategy (**Supplementary Material**), the following exclusion criteria were considered for better selection of the studies: being related to other ocular diseases instead of GD; other imaging approaches than CT or MRI; studies with animals; case reports, reviews, meta-analysis, and treatment outcome investigation studies; and publication in other languages than English.

**Figure 1:**
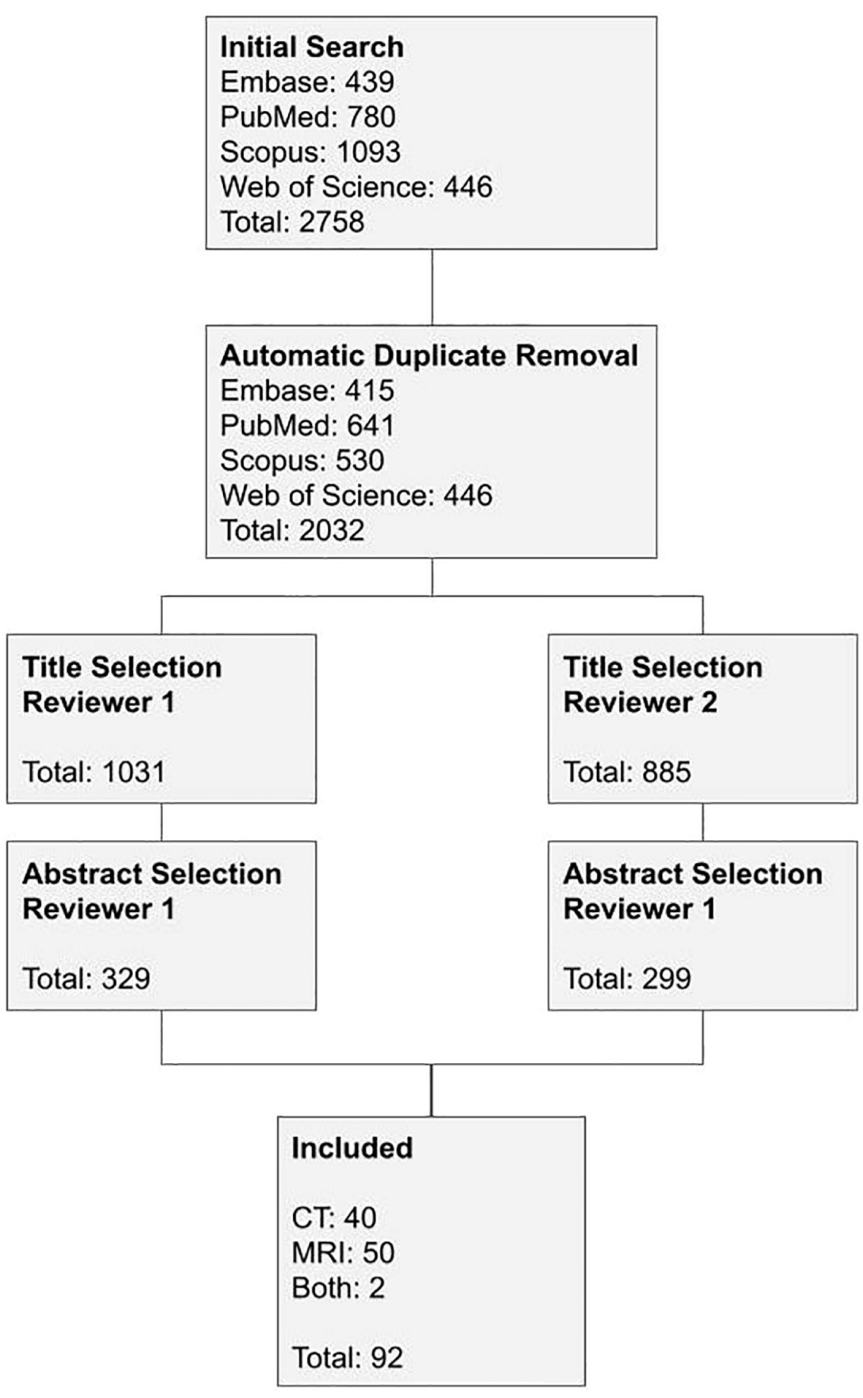
Studies selection flowchart.

All studies were evaluated separately by Rafael Luccas (RL) and Cinthia Minatel Riguetto (CMR), considering the inclusion and exclusion criteria. A total of 2,032 publications were initially retrieved, and 1,703 and 1,733 were excluded by the reviewer RL and CR, respectively, based on the title or abstract. The difference between the selected publications by the reviewers was revised jointly by the reviewers to decide on the inclusion. Three hundred twenty-nine articles were read, of which 234 studies were excluded and 95 studies were considered for the review across CT and MRI modalities, as shown in **Figure 1**.

The following information was retrieved from the publications and summarized in tables, one for each imaging modality detailed: number of subjects, sex, if the subjects were divided into groups on the study, use of control group, mean age with standard deviation, age range, evaluation area, acquisition details (thickness in mm for CT studies and magnetic field intensity in T for MRI studies), evaluation method, software used for imaging analysis, if the proposed method was able to evaluate GO activity or severity, and if the study used any classification for the disease activity.

The study was approved by the local ethical committee (CAAE number 92689218.8.0000.5404).

### Computed tomography data

There were 42 publications included on the CT modality from 1979 to 2020 (**Table 1**). A total of 3,228 subjects were assessed, of which 1,973 were women, representing 61.12% of the sample, whereas male subjects represented 27.14%. In only three studies, the number of male subjects surpassed the number of women. Some studies did not specify the patients’ sex; therefore, 21.43% of the sample is undefined regarding this category. The age of the subjects ranged from 5 to 89 years old, but only 19.05% of the studies included the juvenile population. Of the studies included in this modality, only 38.10% had a control group, and three subclassified the subjects regarding GO activity [e.g., active thyroid eye disease (TED), inactive TED, and GD without ophthalmopathy].

**Table 1:** Studies that used CT as an imaging method to investigate thyroid eye disease.

Artigo		Population					Age			Evaluation Area	Acquisition Details (Thickness in mm)	Method	Software	The proposed method was able to evaluate GO activity or severity?	CAS
First Author	Year	Total	M	F	Details Into Groups	Use Control Group	Mean ± SD	Range							
Alp (19)	2000	111	42	69	Y	Y	41.9 ± 13.9	–		OG, EOM	3	Proptosis, volumetry	–	Y	N
Bingham (20)	2014	125	27	98	N	N	55	18	89	LG	2	Volumetry	OsiriX	Y	N
Bingham (21)	2016	68	–	–	N	N	–	–		OG	1-2	Position	OsiriX	Y	N
Birchall (22)	1996	50	–	–	N	Y	43	24	61	EOM, OG, LG, ON	3	MI	–	Y	N
Byun (23)	2017	80	30	50	Y	Y	–	–		EOM, OF, LG	1	Volumetry, density	–	Y	Y
Byun (24)	2018	48	5	43	N	N	37.44 ± 10.17	20	59	EOM	1	Volumetry	Aquarius iNtuition Client Viewer	Y	Y
Campi (25)	2013	142	38	104	Y	Y	A: 47.6 ± 1.9 B: 56.8 ± 2.9	A: 16 B: 31 T: 16	A: 78 B: 81 T: 78	EB	2	Orbital area	–	Y	Y
Chaganti (26)	2016	85	22	63	N	N	–	18	85	EOM, ON, OG	–	Automated structural metric calculation	eXtensible Neuroimaging Archive Toolkit	Y	N
Chaganti (27)	2018	85	–	–	N	N	–	–		EOM, ON, OG	2	Automated structural metric calculation	–	Y	N
Chaganti (28)	2019	85	22	63	N	N	48.9 ± 13.56	18	83	EOM, ON, OG	–	Automated structural metric calculation	eXtensible Neuroimaging Archive Toolkit	Y	N
Chen (29)	1994	101	26	75	N	N	38.8	14	69	EOM	2	Area, thickness	AutoCad 12	Y	N
Choi (30)	2018	41	18	23	N	Y	42.3 ± 13.8	18	80	OG	2	Area	ImageJ	Y	Y
Choudhary (31)	2019	28	6	22	N	Y	56.4 ± 11.0	–		TT	–	Volumetry	IMPAX PACS	Y	N
Dagi (32)	2011	54	8	46	N	N	52	29	84	EOM	3	Diameter	–	N	N
Doric (33)	2017	91	19	72	N	N	49.49 ± 12.02	–		EOM	–	Proptosis, diameter	–	Y	Y
Fang (34)	2013	325	153	172	N	N	46	05	80	EOM, OF	2.5	Changes	–	Y	N
Feldon (35)	1982	8	2	8	N	Y	–	–		EOM	–	Volumetry	–	Y	Feldon and Unsold
Feldon (36)	1985	49	9	40	N	N	49	11	81	EOM, OF	5 (4 patients) 1.5 (40 patients) 1.0 (5 patients)	Volumetry	–	Y - EOM N - OF	N
Fledelius (37)	1990	28	7	21	N	N	–	24	78	EOM	–	Thickness	–	Y	N
Forbes (38)	1983	19	–	–	N	Y	–	–		EOM, OF	1.5	Volumetry	–	Y	N
Giaconi (39)	2002	12	–	–	N	Y	–	–		EOM, ON, OG	3	Crowding, MI, fat prolapse, proptosis	–	Y	N
Given-Wilson (40)	1989	20	4	16	N	Y	–	26	59	EOM, OG	2 (16 patients) 4 (4 patients)	Muscle index, proptosis	–	Y	N
Wing (41)	1979	12	–	–	N	N	–	–		EOM	5 8	Thickness	–	Y	N
Gonçalves (42)	2012	56	20	36	N	N	–	–		EOM, OF	0.75 1.5 0.7	Crowding	–	Y	N
Gonçalves (43)	2012	61	24	37	N	N	–	–		EOM. OF	–	Volumetry	–	Y	N
Guo (44)	2018	50	24	26	N	N	47	21	68	OG	0.75	Proptosis	iPlan CMF v 3.0	Y	Y
Goodall (45)	1995	24	–	–	N	N	–	–		EOM	3.	Volumetry	–	Y	N
Harris (46)	2012	128	27	101	N	N	–	18	87	LG	–	Volumetry	OsiriX	N	VISA
Huh (47)	2020	126	39	87	N	N	38.2 ± 13.5	15	79	OG	1	Proptosis	PC-based 3D reconstruction software	Y	N
Kim (48)	2015	67	51	16	N	N	40.3 ± 14.6	–		EOM, OF	2	Area	–	Y	Y
Lee (49)	2016	29	15	14	N	Y	54.1 ± 11.4	27	74	EOM	3	Volumetry	ImageJ	Y	N
Le Moli (50)	2012	32	13	19	N	Y	48	22	76	EOM, OG	1.25	Thickness, area	AutoCad	Y	Y
Monteiro (51)	2008	36	12	24	N	N	–	–		EOM	1.5	Diameter	–	Y	Barrets
Murakami (52)	2001	573	118	455	N	N	41	9	80	EOM	2	Thickness	–	Y	N
Nugent (53)	1990	71	19	52	N	Y	50.6	13	79	EOM	–	Diameter	–	Y	N
Ozgen (54)	1999	87	32	55	N	Y	43	16	71	EOM, ON	3	Density, volumetry, position, width	–	Y	N
Polito (55)	1995	16	9	7	N	N	48.75	28	70	EOM	5	Thickness	–	Y	Werner
Potgieser (56)	2019	25	3	22	N	N	48.8	43	62	EOM, OF	–	Volumetry	–	Y	Y
Regensburg (57)	2011	95	–	–	N	Y	–	–		EOM, OF	1.3	Density	Mimics	Y	NOSPECS
Starks (58)	2019	19	6	13	N	N	58	28	77	EOM	0.6	Thickness	Advantage workstation	Y	N
Thornton (59)	2016	16	–	–	N	Y	48 ± 12	–		OF	0.75	Thickness	–	Y	N
Ugradar (60)	2019	50	26	24	N	N	–	–		ON	1	Length	Mimics	N	N

M, Male; F, Female; Y, Yes; N, No; SD, standard deviation; EOM, extraocular muscle; OG, ocular globe; LG, lacrimal gland; ON, optic nerve; OF, orbital fat; mm, millimeters; CAS, clinical activity score; VISA, vision, inflammation, strabismus, and appearance/exposure; NOSPECS, no signs or symptoms, only signs or symptoms, soft tissue involvement, proptosis, extraocular muscle involvement, corneal involvement, and Sight loss.

Different ocular regions were evaluated by these studies, such as extraocular muscles (78.57%), ocular globe (30.95%), orbital fat (23.81%), optic nerve (16.67%), and lacrimal gland (9.52%). Different methods were also applied to assess these areas, including volumetry (30.95%); thickness (19.05%); proptosis (14.29%); area and diameter (9.52% each); automated structural metric calculation (7.14%); position, muscle index, and crowding (4.76% each); and density, changes in general, fat prolapse, diffusion tensor imaging, width, and length (2.38% each).

For CT studies, the acquisition details more frequently reported was the slice thickness, which ranged from 0.6 to 8 mm, with a mean thickness of 3 mm. Ten studies did not specify acquisition details. Only 38.10% of the studies reported the software used for imaging analysis, not considering the publications that indicated that the analysis was done on the workstation.

The study’s proposed methods were able to evaluate GO activity or severity, with 95.24% of them considering that the method demonstrated a promising way of diagnosing or assessing the disease. Only 9.52% reporting the results were not satisfying. A total of 21.43% of the studies evaluated disease activity through the Clinical Activity Score (CAS), whereas 11.90% used other classifications [NOSPECS (no signs or symptoms, only signs or symptoms, soft tissue involvement, proptosis, extraocular muscle involvement, corneal involvement, and sight loss), Werner, Barrets, VISA (vision, inflammation, strabismus, and appearance/exposure), and Feldon and Unsold], and 66.67% did not use any method for disease activity classification.

### Magnetic resonance imaging data

Fifty-two publications from 1988 to 2021 were included in the MRI group (**Table 2**). One thousand nine hundred forty-two subjects were analyzed, of which 1,253 were women, and 591 were men, representing 64.52% and 30.43% of the sample, respectively. In only four studies, the number of male subjects surpassed the number of women. Like in the CT studies, some of them did not specify the patients’ sex; therefore, 5.05% of the sample was undefined. The age of the subjects ranged from 4 to 83 despite 53.85% of them not specifying the age range. Only 7.69% of the studies included the juvenile population. Of the studies included, 67.31% had a control group, and 25% subclassified the subjects according to disease activity (e.g., active TED, inactive TED, and GD without ophthalmopathy).

**Table 2:** Studies that used MR as an imaging method to investigate thyroid eye disease.

Artigo		Population					Age			Evaluation Area	MRI Field (T)	Method	Software/ Toolbox	The proposed method was able to evaluate GO activity or severity?	CAS
First Author	Year	Total	M	F	Details Into Groups	Control Group	Mean ± SD	Range							
Antoniazzi (61)	2004	26	5	21	N	N	10.49 ± 2.61	4	14	EOM, OF	1.5	Volumetry	–	Y	N
Aydin (62)	2003	40	22	18	N	Y	39	21	62	EOM	1.0 1.5	Volumetry	–	Y	N
Bontzos (63)	2019	54	25	29	N	N	57.78 ± 14.71	23	82	OG	1.5	Volumetry	3D Slicer v.4.7.0	N	N
Cakirer (64)	2004	15	7	11	N	Y	41.3	22	51	EOM	1.5	Edema evaluation	–	Y	Werner and Mourits
Cevik (65)	2021	36	13	23	N	Y	40 ± 13	19	75	EOM	1.5	Diameter, proptosis, SIR	–	N	Y
Chen (66)	2020	35	14	21	Y	Y	47.3 ± 14.9	–		EOM, ON	3.0	DTI	DSI Studio software	Y	Y
Chen (67)	2020	40	16	24	Y	N	42.9 ± 14.5	–		EOM	3.0	SIR	Syngo Via	Y	Y
Chen (68)	2020	32	8	24	Y	N	46.1 ± 15.0	–		EOM	3.0	Relaxation time	–	Y	Y
Chen (69)	2021	30	12	18	Y	Y	46.4 ± 13.4	–		LG	3.0	DTI	Syngo Via	Y	Y
Comerci (70)	2013	12	2	10	N	Y	47.2 ± 10.4	–		OF	1.5	Volumetry	–	Y	Y
Dodds (71)	2009	27	–	–	N	Y	40	32	56	EOM, ON	0.5	Diameter, volumetry, proptosis	–	Y	N
Firbank (72)	2000	7	1	6	N	Y	39.9	32	51	EOM	1.0	Volumetry	–	Y	N
Gagliardo (73)	2020	32	10	22	Y	N	49.5	30	68	LG	1.5	Proptosis, herniation	–	Y	Y
Han (74)	2016	20	–	–	N	Y	–	–		EOM	3.0	DTI	OsiriX + DTIMap	Y	Y
Goodall (45)	1995	10	–	–	N	N	–	–		EOM	0.5	Volumetry	–	Y	N
Hiwatashi (75)	2018	23	6	17	Y	N	55.8 ± 12.6	26	83	EOM	3.0	Diffusion	ImageJ	Y	Y
Hosten (76)	1989	39	4	35	N	Y	–	17	79	EOM	0.5	Volumetry	–	Y	N
Hu (77)	2016	33	12	21	N	Y	48.4 ± 13.9	–		LG	3.0	Volumetry, length, width	–	Y	Y
Jiang (78)	2012	34	19	15	Y	Y	46.06 ± 6.63	32	55	EOM	3.0	Dynamic contrast-enhanced imaging	–	Y	Y
Kaichi (79)	2019	22	4	18	N	Y	51	30	82	OF	3.0	Water fraction, volumetry, proptosis	–	Y	Y
Keene (80)	2020	6	3	3	N	Y	46.7 ± 14.0	–		EOM	7.0	Volumetry	–	Y	Y
Khan (81)	2020	110	42	68	N	N	–	–		EOM	–	Thickness	–	Y	N
Kilicarslan (82)	2015	35	9	26	N	Y	40.31 ± 13.50	–		EOM	1.5	Diffusion	–	Y	Y
Kirsch (83)	2010	36	5	31	N	Y	43	15	64	EOM	1.5	SIR	–	Y	Y
Kvetny (84)	2006	15	0	15	N	N	44 ± 9	–		EOM	1.0	Volumetry	ALICE	Y	Y
Lee (85)	2018	20	8	12	N	Y	43.5 ± 10	–		EOM, ON	3.0	DTI, diameter	OsiriX+DTIMap	Y	Y
Lennerstrand (86)	2007	38	5	33	Y	Y	–	–		EOM	1.5	Volumetry	Advantage Windows	Y	N
Majos (87)	2007	45	6	39	N	N	55	19	72	EOM	1.5	Diameter, volumetry, numerical image segmentation	ITK Library	Y	N
Majos (88)	2007	45	6	39	N	N	55	19	72	EOM	1.5	Volumetry, numerical image segmentation	ITK Library	Y	N
Majos (89)	2007	20	8	12	N	N	55	33	61	EOM	1.5	Volumetry, numerical image segmentation, SIR, relaxation time	–	Y	N
Matsuzawa (90)	2020	47	17	30	Y	Y	–	–		EOM	3.0	SIR	syngo MapIt	Y	Y
Nishida (91)	2001	6	3	3	Y	Y	64 ± 9.7	–		EOM, OF	1.5	Thickness, volumetry	–	Y	N
Nishida (92)	2002	10	8	2	N	Y	57.0 ± 14.0	–		EOM, OF	1.5	Volumetry	Video 2.0	Y	N
Ohnishi (93)	1993	53	15	38	Y	Y	–	–		EOM	0.5	Thickness	–	Y	Werner
Ohnishi (94)	1994	110	30	80	Y	Y	–	–		EOM	0.5	Relaxation time	–	Y	Werner
Ollitrault (95)	2021	206	71	135	N	N	52.3 ± 13.2	–		EOM, OF	3.0	Inflammation, volumetry, degeneration, fibrosis	Carestream Vue PACS	Y	Y
Ozkan (96)	2015	28	8	20	N	Y	45.96 ± 13.75	25	68	ON	3.0	DTI	–	Y	Y
Politi (97)	2014	74	25	49	N	Y	52 ± 13	–		EOM	1.5	Diffusion	–	Y	Y
Polito (55)	1995	16	9	7	N	N	48.75	28	70	EOM	0.5	Thickness	–	Y	Werner
Razek (98)	2017	33	16	17	N	Y	36 ± 12.2	18	55	EOM	1.5	Diffusion	–	Y	Y
Razek (99)	2019	44	17	27	N	Y	38 ± 12.6	18	52	LG	1.5	Diffusion	–	Y	Y
Rodríguez-González (100)	2011	28	4	24	N	Y	47.5 ± 11.53	26	72	EOM, OF	1.5	SIR	ViewForum R5.1V1L1 SP1	Y	Y
Rutkowska-Hinc (101)	2018	37	12	25	N	N	–	–		EOM, OG	1.5	Crowding, length, MI	–	Y	Y
Shen (102)	2018	10	3	7	N	Y	34.1 ± 7.1	24	41	EOM, OF	1.5	Volumetry	Mimics	Y	Y
Silkiss (103)	2016	10	0	10	N	Y	–	–		BR	–	Thickness	FSL Toolbox	Y	Y
So (104)	2000	37	4	33	N	N	–	–		EOM	1.5	MI	–	Y	N
Szucs-Farkas (105)	2002	35	6	29	N	Y	49.3	28	79	EOM	1.0	Diameter	–	Y	N
Taoka (106)	2005	16	–	–	N	Y	–	16	54	EOM	1.0	Peak enhancement ratio	–	Y	N
Tortora (107)	2013	16	7	9	N	Y	49.19	–		EOM	1.5	SIR	–	Y	Y
Troelsta (108)	1988	18	–	–	N	N	–	–		EOM	1.5	Volumetry	–	Y	NOSPECS
Wu (109)	2021	111	33	68	Y	Y	–	–		LG	3.0	Area, length, width, diffusion, relaxation time	Tissue 4D.	Y	Y
Wu (110)	2021	60	31	29	N	Y	–	–		ON, CSF	3.0	Diameter, area, water fraction, volumetry	–	Y	Y

M, male; F, female; Y, yes; N, no; SD, standard deviation; EOM, extraocular muscle; OG, ocular globe; LG, lacrimal gland; ON, optic nerve; OF, orbital fat; BR, brain; CSF, cerebrospinal fluid; T, tesla; MI, muscle index; DTI, diffusion tensor imaging; SIR, signal intensity ratio; CAS, clinical activity score; NOSPECS, no signs or symptoms, only signs or symptoms, soft tissue involvement, proptosis, extraocular muscle involvement, corneal Involvement, and sight loss.

Different ocular regions were assessed, such as extraocular muscles (78.85%), orbital fat (15.38%), lacrimal gland and optic nerve (9.62% each), ocular globe (3.85%), and brain and cerebrospinal fluid (1.92% each). Different methods were also applied to evaluate these areas, including volumetry (40.38%); diameter, diffusion, and signal intensity ratio (11.54% each); diffusion tensor imaging (9.62%); proptosis, thickness, and relaxation time (7.69% each); length and numerical image segmentation (5.77% each); muscle index, area, width, and water fraction (3.85% each); and crowding, edema, herniation, dynamic contrast-enhanced imaging, inflammation, degeneration, fibrosis, and peak enhancement ratio (1.92% each).

The acquisition detail, more frequently, was the magnetic field, ranging from 0.5 T to 7.0 T, with a mean field of 1.5 T. Only 34.62% of the studies reported the software used for imaging analysis, not considering the publications that indicated that the analysis was done on the workstation.

The study’s proposed methods were able to evaluate GO activity or severity, with 96.15% considering the used method demonstrated a promising way of diagnosing or evaluating the disease. Only 3.85% reported that the results were not satisfying. The disease activity was assessed through CAS by 59.62% of the studies, whereas 9.62% used other classifications (NOSPECS and Werner), and 32.69% did not use any method for disease classification.

## Discussion

GO can be diagnosed at different stages, and it is crucial to identify the stage in order to guide treatment approaches (111). Unfortunately, only a small percentage of the studies included herein identified the stages and evaluated them according to subgroups. Studies seeking to evaluate diagnostic methods and disease grading would better consider disease stage to evidence which method is more sensitive for each distinct stage, as shown by Chen et al. (65–69). In this MRI study, all findings suggested that the methods used can help to identify the disease activity and staging, providing a better perspective on treatment options. Gagliardo et al. (73) also evaluated the subjects into different groups regarding the disease activity, and their findings pointed to an easy method for daily practice to diagnose and identify the disease stage.

Across the included studies, there were seven areas investigated. In the CT studies, the investigators analyzed the extraocular muscle, the ocular globe, the lacrimal gland, the optic nerve, and the orbital fat. The extraocular muscles were the most investigated area, with 76.74% of the studies analyzing it, mostly its volumetry, thickness, and diameter because it is reported as the most affected area by the disease (112) and considered an important factor to determine the disease diagnosis and its activity. Han et al. (74), different from other studies, used diffusion tensor imaging to measure the fractional anisotropy, mean, axial, and radial diffusivities of the muscles. The authors not only stated that diffusion imaging allows the identification of the disease stage, as acute or chronic, but also recognized its limitations on reflecting the disease activity according to the CAS. Dagi et al. (32) and Su et al. (15) found a weak correlation between extraocular muscle diameters and motility in younger subjects but stronger in older patients with active TED. The studies varied in affirming which extraocular muscle is the most affected by the disease, if the inferior rectus muscle (19), the medial (36) or lateral rectus muscles (37), but all agree on muscle involvement in general. Potgieser et al. (56) also found that the extraocular muscle involvement decreases in regard to volume over the years of natural disease history, inversely proportional to orbital fat that increases the volume over the years. Orbital fat was also investigated by CT (23.26%), and as related to ophthalmopathy as the extraocular muscles. Feldon et al. (36) state that the orbital fat does not play an essential role in the disease; however, others (31, 34, 39, 57) demonstrated the relation of the orbital fat with the disease progress and the importance on imaging findings to diagnose and evaluate this condition, by measuring the orbital fat density, ratio with orbital area, or volume. The ocular globe was mostly assessed by its position and proptosis or better methods to do these measures (44), also correlating the extraocular muscles and orbital fat involvement (19, 21).

Harris et al. (46) found lacrimal gland enlargement in GO with a weak correlation with proptosis or inflammation, contested by Bingham et al. (20), who affirmed that the lacrimal gland enlargement was positively correlated with proptosis.

MRI studies investigated the extraocular muscle, the ocular globe, the lacrimal gland, the optic nerve, the orbital fat, the cerebrospinal fluid, and the brain. Similarly to the CT studies, the extraocular muscles were evaluated by 78.85% of the studies using mainly volumetry, diameter, thickness, diffusion (weighted and tensor imaging), and signal intensity ratio. The findings of volumetry, diameter, and thickness were very similar to the ones with CT; however, the use of MRI allows the investigator to approach the disease with other methods, such as diffusion and signal intensity ratio, used together by 32.69% of the studies. Diffusion tensor imaging studies (66, 69) can assess microstructural changes in the extraocular muscles and indicate the disease activity with mean diffusivity metrics of medial extraocular muscles, whereas Han et al. (74) agreed with the advantages of this method remarking that the fractional anisotropy and radial diffusivity are better markers for the disease activity. The diffusion-weighted imaging was evaluated (82, 98) using the apparent diffusion coefficient as a metric on the extraocular muscles stating to diagnosis. The study highlights that the medial rectus muscle is the better one to obtain the metric, and the study by Kilicarslan et al. (82) found that the metric also correlates to ophthalmologic tests, making the method a promising option because the diffusion-weighted imaging sequence can be obtained on routine MRI, improving information about the disease. The signal intensity ratio of T1 and T2 images was also promising in indicating disease activity (83, 97). Both studies pointed out the method used to indicate disease activity in extraocular muscles, helping to determine the best treatment approach, because T1 and T2 images are sequences collected routinely.

Orbital fat findings in MRI are similar to the findings in CT studies (92, 102). The degree of exophthalmos and the volume rate obtained with MRI were both accurate and reliable. The sum of all the findings regarding orbital fat indicates that not only the extraocular muscles but also the orbital fat should be routinely when evaluating GO. The optic nerve and lacrimal gland were assessed by MRI mainly by diffusion methods. Chen et al. (66), Lee et al. (85), and Ozkan et al. (96) used diffusion tensor imaging to evaluate the optic nerve indicating fractional anisotropy and mean diffusivity as the main metrics to assess the nerve, whereas Chen et al. (69) used the method to evaluate the lacrimal gland, showing that the metrics, particularly the fractional anisotropy, can be useful to reveal the disease activity. Razek et al. (99) and Wu et al. (109) also evaluated the lacrimal gland but using diffusion-weighted imaging and signal intensity ratio, respectively, which both demonstrated promising results for diagnosing and predicting disease activity.

Interestingly, two studies investigated unusual areas of TED: brain and cerebrospinal fluid. Wu et al. (110) evaluated the volume of cerebrospinal fluid in the optic nerve sheath by applying a semi-automatic segmentation algorithm based on water images and stated that the cerebrospinal fluid was causing the enlargement of the optic nerve sheath, concluding that these measurements were sensitive for dysthyroid optic neuropathy. Silkiss et al. (103) evaluated the brain anatomy in patients with thyroid ophthalmopathy using an automatic cortical segmentation algorithm to estimate the thickness of the subject’s grey matter and found changes in six locations on the right hemisphere and two on the left hemisphere, possibly associated with the cognitive changes reported by the patients. These two studies showed that new approaches not only in methods but also on sites of investigation could be very promising for a better understanding and management of GO.

Most methods have not achieved sufficient scientific evidence regarding their reproducibility. Studies performed with CT were the least specific when describing the acquisition methods, 23.26% of them did not specify the image acquisition details, and others mostly indicated the thickness of the slices. Otherwise, MRI studies showed the field potency, highly relevant due to this information directly reflecting on the quality of the acquired image and, consequently, the quality of the outcomes. Thus, regarding reproducibility, only a few studies (36.84%) across CT and MRI reported the software used for the image analysis, and they were different among them, indicating that, despite the method used, there is no standard procedure for evaluating the disease when it comes to imaging analysis of non-dimensional metrics.

One of the most critical factors to validate a method was the correlation with the assessments already established as standard procedures in medical practice, such as CAS when it comes to TED. In CT studies, 65.12% did not use any disease activity assessments, whereas, in MRI studies, the percentage found was 32.69%. The use of such assessments in clinical studies to determine their validity and to increase the chance for the method to become implemented in the hospital routine is relevant.

## Conclusion and future directions

This review demonstrated that, despite the fact that several studies have been performed using different methods and approaches for TED evaluation, there is still a lack of standardization of measurements and outcome reports. Indeed, important information related to method implementation or reproducibility and specifications about the software used for analysis or the imaging acquisition parameter have not been reported. Enhancement of reproducibility and methodological heterogeneity will set the stage for more robust studies to define its clinical relevance. As discussed before, there is also a need for studies about TED that relate common practice and disease management to achieve more effective and realistic measurements and direct treatment strategies.

Regarding the scope of the studies, there was a considerable discrepancy between the ones evaluating extraocular muscles and those evaluating other areas. Orbital fat comes right after the extraocular muscles, but these studies showed that other areas also have promising results when it comes to diagnosing and identifying the disease activity, highlighting the studies with lacrimal gland, optic nerve, and the brain.

Considering the study designs, even using different methods, several attention points should be considered. For more homogeneous, reproducible, and reliable study designs, we propose that:

- the investigators consider using control groups to increase the reliability of the results because the disease diagnosis is the focus and should be, whenever possible, compared to healthy subjects for a better understanding of the alterations between groups;
- the acquisition of the image be best possible within the study site capabilities for a guaranteed quality of data obtained on image analysis;
- relate the results with scores of approved and validated assessment methods such as CAS for the GO;
- better description of the study design, methods, and data or image acquisition on the published papers, indicating, for example, which software was used for imaging analysis and statistical results, acquisition protocols, metrics for imaging, and equipment details.

In the revised studies, it was possible to notice that most of the methods used still need to be corroborated and validated; therefore, more studies should be performed to include these methods in clinical practice, facilitating the diagnosis and approach for the treatment of TED.

## Author contributions

RL: Conceptualization, Data curation, Formal Analysis, Methodology, Visualization, Writing – original draft, Writing – review & editing. CMR: Validation, Writing – review & editing. MA: Conceptualization, Writing – review & editing. DEZ-W: Conceptualization, Writing – review & editing. FR: Conceptualization, Methodology, Project administration, Supervision, Writing – review & editing.
