# Supplementary material for: Computed tomography and magnetic resonance imaging approaches to Graves’ ophthalmopathy: a narrative review
Source: Front Endocrinol (Lausanne). 2024 Jan 8;14:1277961. doi: 10.3389/fendo.2023.1277961 (PMC10801040; doi:10.3389/fendo.2023.1277961)
Supplement: Supplementary file 1 [file Table_1.docx]

| **Databases** | **Strategies** |
| --- | --- |
| **Pubmed** | ((((((((((((((((((((((((((((((((((("Graves Ophthalmopathy"[MeSH Terms]) OR ("Ophthalmopathy, Graves"[MeSH Terms])) OR ("Ophthalmopathies, Thyroid-Associated"[MeSH Terms])) OR ("Ophthalmopathies, Thyroid Associated"[MeSH Terms])) OR ("Thyroid-Associated Ophthalmopathies"[MeSH Terms])) OR ("Thyroid Associated Ophthalmopathies"[MeSH Terms])) OR ("Dysthyroid Ophthalmopathy"[MeSH Terms])) OR ("Dysthyroid Ophthalmopathies"[MeSH Terms])) OR ("Ophthalmopathies, Dysthyroid"[MeSH Terms])) OR ("Ophthalmopathy, Dysthyroid"[MeSH Terms])) OR ("Thyroid-Associated Ophthalmopathy"[MeSH Terms])) OR ("Thyroid Associated Ophthalmopathy"[MeSH Terms])) OR ("Graves Orbitopathy"[MeSH Terms])) OR ("Graves Orbitopathies"[MeSH Terms])) OR ("Orbitopathies, Graves"[MeSH Terms])) OR ("Orbitopathy, Graves"[MeSH Terms])) OR ("Ophthalmopathy, Thyroid-Associated"[MeSH Terms])) OR ("Ophthalmopathy, Thyroid Associated"[MeSH Terms])) OR ("Myopathic Ophthalmopathy"[MeSH Terms])) OR ("Myopathic Ophthalmopathies"[MeSH Terms])) OR ("Ophthalmopathies, Myopathic"[MeSH Terms])) OR ("Ophthalmopathy, Myopathic"[MeSH Terms])) OR ("Congestive Ophthalmopathy"[MeSH Terms])) OR ("Congestive Ophthalmopathies"[MeSH Terms])) OR ("Ophthalmopathies, Congestive"[MeSH Terms])) OR ("Ophthalmopathy, Congestive"[MeSH Terms])) OR ("Edematous Ophthalmopathy"[MeSH Terms])) OR ("Edematous Ophthalmopathies"[MeSH Terms])) OR ("Ophthalmopathies, Edematous"[MeSH Terms])) OR ("Ophthalmopathy, Edematous"[MeSH Terms])) OR ("Ophthalmopathy, Infiltrative"[MeSH Terms])) OR ("Infiltrative Ophthalmopathies"[MeSH Terms])) OR ("Infiltrative Ophthalmopathy"[MeSH Terms])) OR ("Ophthalmopathies, Infiltrative"[MeSH Terms])) OR (((((((((((((((((((((((((((((((((("Graves Ophthalmopathy"[Title/Abstract]) OR ("Ophthalmopathy Graves"[Title/Abstract])) OR ("Ophthalmopathies Thyroid-Associated"[Title/Abstract])) OR ("Ophthalmopathies Thyroid Associated"[Title/Abstract])) OR ("Thyroid-Associated Ophthalmopathies"[Title/Abstract])) OR ("Thyroid Associated Ophthalmopathies"[Title/Abstract])) OR ("Dysthyroid Ophthalmopathy"[Title/Abstract])) OR ("Dysthyroid Ophthalmopathies"[Title/Abstract])) OR ("Ophthalmopathies, Dysthyroid"[Title/Abstract])) OR ("Ophthalmopathy Dysthyroid"[Title/Abstract])) OR ("Thyroid-Associated Ophthalmopathy"[Title/Abstract])) OR ("Thyroid Associated Ophthalmopathy"[Title/Abstract])) OR ("Graves Orbitopathy"[Title/Abstract])) OR ("Graves Orbitopathies"[Title/Abstract])) OR ("Orbitopathies Graves"[Title/Abstract])) OR ("Orbitopathy Graves"[Title/Abstract])) OR ("Ophthalmopathy Thyroid-Associated"[Title/Abstract])) OR ("Ophthalmopathy Thyroid Associated"[Title/Abstract])) OR ("Myopathic Ophthalmopathy"[Title/Abstract])) OR ("Myopathic Ophthalmopathies"[Title/Abstract])) OR ("Ophthalmopathies Myopathic"[Title/Abstract])) OR ("Ophthalmopathy Myopathic"[Title/Abstract])) OR ("Congestive Ophthalmopathy"[Title/Abstract])) OR ("Congestive Ophthalmopathies"[Title/Abstract])) OR ("Ophthalmopathies, Congestive"[Title/Abstract])) OR ("Ophthalmopathy, Congestive"[Title/Abstract])) OR ("Edematous Ophthalmopathy"[Title/Abstract])) OR ("Edematous Ophthalmopathies"[Title/Abstract])) OR ("Ophthalmopathies Edematous"[Title/Abstract])) OR ("Ophthalmopathy Edematous"[Title/Abstract])) OR ("Ophthalmopathy, Infiltrative"[Title/Abstract])) OR ("Infiltrative Ophthalmopathies"[Title/Abstract])) OR ("Infiltrative Ophthalmopathy"[Title/Abstract])) OR ("Ophthalmopathies Infiltrative"[Title/Abstract]))) **AND** ((((((((((((((((((((((((((((((((((((((((((("Magnetic Resonance Imaging"[MeSH Terms]) OR ("Imaging, Magnetic Resonance"[MeSH Terms])) OR ("NMR Imaging"[MeSH Terms])) OR ("Imaging, NMR"[MeSH Terms])) OR ("Tomography, NMR"[MeSH Terms])) OR ("Tomography, MR"[MeSH Terms])) OR ("MR Tomography"[MeSH Terms])) OR ("NMR Tomography"[MeSH Terms])) OR ("Steady-State Free Precession MRI"[MeSH Terms])) OR ("Steady State Free Precession MRI"[MeSH Terms])) OR ("Zeugmatography"[MeSH Terms])) OR ("Imaging, Chemical Shift"[MeSH Terms])) OR ("Chemical Shift Imagings"[MeSH Terms])) OR ("Imagings, Chemical Shift"[MeSH Terms])) OR ("Shift Imaging, Chemical"[MeSH Terms])) OR ("Shift Imagings, Chemical"[MeSH Terms])) OR ("Chemical Shift Imaging"[MeSH Terms])) OR ("Magnetic Resonance Image"[MeSH Terms])) OR ("Image, Magnetic Resonance"[MeSH Terms])) OR ("Magnetic Resonance Images"[MeSH Terms])) OR ("Resonance Image, Magnetic"[MeSH Terms])) OR ("Magnetization Transfer Contrast Imaging"[MeSH Terms])) OR ("MRI Scans"[MeSH Terms])) OR ("MRI Scan"[MeSH Terms])) OR ("Scan, MRI"[MeSH Terms])) OR ("Scans, MRI"[MeSH Terms])) OR ("Tomography, Proton Spin"[MeSH Terms])) OR ("Proton Spin Tomography"[MeSH Terms])) OR (fMRI[MeSH Terms])) OR ("MRI, Functional"[MeSH Terms])) OR ("Functional MRI"[MeSH Terms])) OR ("Functional MRIs"[MeSH Terms])) OR ("MRIs, Functional"[MeSH Terms])) OR ("Functional Magnetic Resonance Imaging"[MeSH Terms])) OR ("Magnetic Resonance Imaging, Functional"[MeSH Terms])) OR ("Spin Echo Imaging"[MeSH Terms])) OR ("Echo Imaging, Spin"[MeSH Terms])) OR ("Echo Imagings, Spin"[MeSH Terms])) OR ("Imaging, Spin Echo"[MeSH Terms])) OR ("Imagings, Spin Echo"[MeSH Terms])) OR ("Spin Echo Imagings"[MeSH Terms])) OR ((((((((((((((((((((((((((((((((((((((((("Magnetic Resonance Imaging"[Title/Abstract]) OR ("Imaging, Magnetic Resonance"[Title/Abstract])) OR ("NMR Imaging"[Title/Abstract])) OR ("Imaging, NMR"[Title/Abstract])) OR ("Tomography, NMR"[Title/Abstract])) OR ("Tomography, MR"[Title/Abstract])) OR ("MR Tomography"[Title/Abstract])) OR ("NMR Tomography"[Title/Abstract])) OR ("Steady-State Free Precession MRI"[Title/Abstract])) OR ("Steady State Free Precession MRI"[Title/Abstract])) OR ("Zeugmatography"[Title/Abstract])) OR ("Imaging, Chemical Shift"[Title/Abstract])) OR ("Chemical Shift Imagings"[Title/Abstract])) OR ("Imagings, Chemical Shift"[Title/Abstract])) OR ("Shift Imaging, Chemical"[Title/Abstract])) OR ("Shift Imagings, Chemical"[Title/Abstract])) OR ("Chemical Shift Imaging"[Title/Abstract])) OR ("Magnetic Resonance Image"[Title/Abstract])) OR ("Image, Magnetic Resonance"[Title/Abstract])) OR ("Magnetic Resonance Images"[Title/Abstract])) OR ("Resonance Image, Magnetic"[Title/Abstract])) OR ("Magnetization Transfer Contrast Imaging"[Title/Abstract])) OR ("MRI Scans"[Title/Abstract])) OR ("MRI Scan"[Title/Abstract])) OR ("Scan, MRI"[Title/Abstract])) OR ("Scans, MRI"[Title/Abstract])) OR ("Tomography, Proton Spin"[Title/Abstract])) OR ("Proton Spin Tomography"[Title/Abstract])) OR (fMRI[Title/Abstract])) OR ("MRI, Functional"[Title/Abstract])) OR ("Functional MRI"[Title/Abstract])) OR ("Functional MRIs"[Title/Abstract])) OR ("MRIs, Functional"[Title/Abstract])) OR ("Functional Magnetic Resonance Imaging"[Title/Abstract])) OR ("Magnetic Resonance Imaging, Functional"[Title/Abstract])) OR ("Spin Echo Imaging"[Title/Abstract])) OR ("Echo Imaging, Spin"[Title/Abstract])) OR ("Echo Imagings, Spin"[Title/Abstract])) OR ("Imaging, Spin Echo"[Title/Abstract])) OR ("Imagings, Spin Echo"[Title/Abstract])) OR ("Spin Echo Imagings"[Title/Abstract]))) OR ((("Tomography"[MeSH Terms]) OR ("Tomographies"[MeSH Terms])) OR (("Tomography"[ Title/Abstract]) OR ("Tomographies"[ Title/Abstract])))) |
| **Embase** | ('endocrine ophthalmopathy'/exp OR 'endocrine ophthalmopathy' OR 'myopathic ophthalmopathy' OR 'congestive ophthalmopathy' OR 'edematous ophthalmopathy' OR 'infiltrative ophthalmopathies' OR 'graves ophthalmopathy'/exp OR 'graves ophthalmopathy') AND [embase]/lim NOT ([embase]/lim AND [medline]/lim) AND (('tomography'/exp OR 'tomography') AND [embase]/lim NOT ([embase]/lim AND [medline]/lim) OR (('nuclear magnetic resonance imaging'/exp OR 'nuclear magnetic resonance imaging' OR 'mr tomography' OR 'nmr tomography' OR 'steady-state free precession mri' OR 'steady state free precession mri' OR zeugmatography OR 'chemical shift imaging'/exp OR 'chemical shift imaging' OR 'magnetic resonance image' OR 'magnetization transfer contrast imaging' OR 'mri scan' OR 'proton spin tomography' OR 'fmri'/exp OR fmri OR 'functional mri'/exp OR 'functional mri' OR 'functional magnetic resonance imaging'/exp OR 'functional magnetic resonance imaging' OR 'spin echo imaging'/exp OR 'spin echo imaging') AND [embase]/lim NOT ([embase]/lim AND [medline]/lim))) |
| **Cochrane** | MeSH descriptor: [Graves Ophthalmopathy] explode all trees AND “MeSH descriptor: [Magnetic Resonance Imaging] explode all trees OR MeSH descriptor: [Tomography] explode all trees” |
| **Scopus** | ( TITLE-ABS-KEY ( "Graves Ophthalmopathy" ) OR TITLE-ABS-KEY ( "Thyroid-Associated Ophthalmopathies" ) OR TITLE-ABS-KEY ( "Thyroid Associated Ophthalmopathies" ) OR TITLE-ABS-KEY ( "Dysthyroid Ophthalmopathy" ) OR TITLE-ABS-KEY ( "Thyroid-Associated Ophthalmopathy" ) OR TITLE-ABS-KEY ( "Thyroid Associated Ophthalmopathy" ) OR TITLE-ABS-KEY ( "Graves Orbitopathy" ) OR TITLE-ABS-KEY ( "Myopathic Ophthalmopathy" ) OR TITLE-ABS-KEY ( "Congestive Ophthalmopathy" ) OR TITLE-ABS-KEY ( "Edematous Ophthalmopathy" ) OR TITLE-ABS-KEY ( "Infiltrative Ophthalmopathy" ) ) AND ( ( TITLE-ABS-KEY ( tomography ) ) OR ( TITLE-ABS-KEY ( magnetic AND resonance AND imaging ) OR TITLE-ABS-KEY ( nmr AND imaging ) OR TITLE-ABS-KEY ( "MR Tomography" ) OR TITLE-ABS-KEY ( "NMR Tomography" ) OR TITLE-ABS-KEY ( "Steady-State Free Precession MRI" ) OR TITLE-ABS-KEY ( "Steady State Free Precession MRI" ) OR TITLE-ABS-KEY ( "Zeugmatography" ) OR TITLE-ABS-KEY ( "Chemical Shift Imagings" ) OR TITLE-ABS-KEY ( "Chemical Shift Imaging" ) OR TITLE-ABS-KEY ( "Magnetic Resonance Image" ) OR TITLE-ABS-KEY ( "Magnetic Resonance Images" ) OR TITLE-ABS-KEY ( "Magnetization Transfer Contrast Imaging" ) OR TITLE-ABS-KEY ( "MRI Scans" ) OR TITLE-ABS-KEY ( "Proton Spin Tomography" ) OR TITLE-ABS-KEY ( fmri ) OR TITLE-ABS-KEY ( "Functional MRI" ) OR TITLE-ABS-KEY ( "Functional Magnetic Resonance Imaging" ) OR TITLE-ABS-KEY ( "Spin Echo Imaging" ) ) ) |
| **WebOfScience** | TS=("Graves Ophthalmopathy") OR TI=("Graves Ophthalmopathy") OR TS=("Thyroid-Associated Ophthalmopathies") OR TI=("Thyroid-Associated Ophthalmopathies") OR TS=("Thyroid Associated Ophthalmopathies") OR TI=("Thyroid Associated Ophthalmopathies") OR TS=("Dysthyroid Ophthalmopathy") OR TI=("Dysthyroid Ophthalmopathy") OR TS=("Thyroid-Associated Ophthalmopathy") OR TI=("Thyroid-Associated Ophthalmopathy") OR TS=("Thyroid Associated Ophthalmopathy") OR TI=("Thyroid Associated Ophthalmopathy") OR TS=("Graves Orbitopathy") OR TI=("Graves Orbitopathy") OR TS=("Myopathic Ophthalmopathy") OR TI=("Myopathic Ophthalmopathy") OR TS=("Congestive Ophthalmopathy") OR TI=("Congestive Ophthalmopathy") OR TS=("Edematous Ophthalmopathy") OR TI=("Edematous Ophthalmopathy") OR TS=("Infiltrative Ophthalmopathy") OR TI=("Infiltrative Ophthalmopathy") Índices=SCI-EXPANDED, SSCI, A&HCI, CPCI-S, CPCI-SSH, ESCI Tempo estipulado=Todos os anos Editar AND TS=("Tomography") OR TI=("Tomography") Índices=SCI-EXPANDED, SSCI, A&HCI, CPCI-S, CPCI-SSH, ESCI Tempo estipulado=Todos os anos OR TS=("Magnetic Resonance Imaging") OR TI=("Magnetic Resonance Imaging") OR TS=("NMR Imaging") OR TI=("NMR Imaging") OR TS=("MR Tomography") OR TI=("MR Tomography") OR TS=("NMR Tomography") OR TI=("NMR Tomography") OR TS=("Steady-State Free Precession MRI") OR TI=("Steady-State Free Precession MRI") OR TS=("Steady State Free Precession MRI") OR TI=("Steady State Free Precession MRI") OR TS=("Zeugmatography") OR TI=("Zeugmatography") OR TS=("Chemical Shift Imaging") OR TI=("Chemical Shift Imaging") OR TS=("Magnetic Resonance Image") OR TI=("Magnetic Resonance Image") OR TS=("Magnetization Transfer Contrast Imaging") OR TI=("Magnetization Transfer Contrast Imaging") OR TS=("MRI Scans") OR TI=("MRI Scans") OR TS=("MRI Scan") OR TI=("MRI Scan") OR TS=("Proton Spin Tomography") OR TI=("Proton Spin Tomography") OR TS=(fMRI) OR TI=(fMRI) OR TS=("Functional MRI") OR TI=("Functional MRI") OR TS=("Functional Magnetic Resonance Imaging") OR TI=("Functional Magnetic Resonance Imaging") OR TS=("Spin Echo Imaging") OR TI=("Spin Echo Imaging") Índices=SCI-EXPANDED, SSCI, A&HCI, CPCI-S, CPCI-SSH, ESCI Tempo estipulado=Todos os anos |
